# Role of Phenolic Acid Metabolism in Enhancing Bioactivity of Mentha Extract Fermented with Plant-Derived *Lactobacillus plantarum* SN13T

**DOI:** 10.1007/s12602-023-10103-4

**Published:** 2023-06-06

**Authors:** Shrijana Shakya, Narandalai Danshiitsoodol, Masafumi Noda, Masanori Sugiyama

**Affiliations:** Department of Probiotic Science for Preventive Medicine, Graduate School of Biomedical and Health Sciences, Hiroshima University, Hiroshima, Japan

**Keywords:** *Lactobacillus*, Mentha extract, Rosmarinic acid, Dihydrocaffeic acid, Phenolic acid metabolism

## Abstract

Plant-derived lactic acid bacteria are major fermentation organisms that can grow in medicinal herb extracts enriched with phytochemicals like glycosides, phenolic acids, flavonoids, and tannins. Fermentation with strain-specific *Lactobacilli* harboring metabolic enzymes can increase the bioactivity and bioavailability of medicinal herbs. Fermentation of extracts of *Artemisia princeps* and *Paeonia lactiflora* has been previously found to increase their bioactivities. Therefore, this study explores the possibility of increasing the bioactivity of *Mentha arvensis* (Mentha) extract against lipopolysaccharide (LPS)-induced RAW 264.7 macrophage cells by fermenting with plant-derived probiotic strains *Lactobacillus (Lact.) plantarum* SN13T and *Pediococcus (Ped.) pentosaceus* LP28. As a result, fermentation with SN13T significantly increased the bioactivity of Mentha extract as compared to unfermented or LP28-fermented extracts. This higher bioactivity was associated with the metabolism of rosmarinic acid (RA) and caffeic acid (CA), the major bioactive phenolic acids reported in Mentha, along with the production of the metabolite dihydrocaffeic acid (DHCA). DHCA was found to be a more potent LPS-induced nitric oxide (NO) inhibitor than its precursor phenolic acids. The metabolism of RA to DHCA via CA could be mediated by the enzymes cinnamoyl ester hydrolase and hydroxycinnamate reductases, encoded by the *ceh* gene and the *hcrRABC* gene operon, respectively, which were identified in the complete genome sequence of *Lact. plantarum* SN13T but were absent in *Ped. pentosaceus* LP28. The genes *hcrA*, *hcrB*, and *hcrC* were significantly and time-dependently overexpressed in *Lact. plantarum* SN13T when grown in the Mentha extract, suggesting the role of phenolic acid metabolism in enhancing its bioactivity.

**Supplementary Information:**

The online version contains supplementary material available at 10.1007/s12602-023-10103-4.

## Introduction

Lactic acid bacteria (LAB) are the most important microorganisms that are capable of inducing significant changes in the health-promoting properties of plant foods [1]. Plants, particularly, medicinal herbs, are reservoirs of bioactive compounds like glycosides, antioxidants, phenolics, and dietary fibers, as well as vitamins and minerals, which enable species and strain-specific LAB to follow various metabolic routes [2]. During the fermentation process of plants by LAB, the decomposition and/or biotransformation of complex phytochemicals into compatible components can improve their bioavailability and bioactivity, as well as concentrate the functional microbial metabolites with beneficial consequences for human health [3]. LAB species that mainly dominate spontaneous plant fermentation such as *Lact. plantarum*, *Lact. pentosus*, and *Pediococcus* inherit a dedicated portfolio of enzymes like glycosyl hydrolase, phenolic acid decarboxylase and reductase, and esterase activities that catalyze the conversions of glycosides, phenolic acids, and tannins to biologically active metabolites [1].

Over 1200 strains of plant-derived LAB from fruits, vegetables, flowers, and medicinal plants have been isolated and evaluated for their health benefits by our research group. Notably, *Lactobacillus plantarum* SN13T, which has been isolated from banana leaves, was found to improve liver function by altering the composition of gut microbiota in subjects with mild liver dysfunction [4]. *Pediococcus pentosaceus* LP28, isolated from the longan fruit *Euphoria longana*, reduced obesity and fatty liver in high-fat diet-induced mice and reduced BMI, body fat, and waist circumference, suggesting that it is a promising anti-obesity candidate for preventing metabolic syndrome [5]. Furthermore, when *Lact. plantarum* SN13T or *Lact. brevis* 174A was grown in medicinal herb extracts like *Artemisia princeps* Pampanini and *Paeonia lactiflora* Pall, bioactive compounds were produced that have improved their therapeutic potential [6-8]. Therefore, we speculated that the bioactivity of the medicinal herb, *Mentha arvensis* Linné var. *piperascens* Malinvaud (Lamiaceae), i.e., Mentha, can also be enhanced by fermentation with plant-derived LAB.

Mentha, among the most popular herbs, is widely used in cooking and cosmetics. Traditionally, it has been used for the treatment of gastrointestinal disorders such as flatulence, indigestion, nausea, vomiting, anorexia, and ulcerative colitis. Moreover, the essential oil and extracts of Mentha species have been reported to possess antimicrobial, fungicidal, antiviral, insecticidal, and antioxidant properties [9]. Apart from essential oils such as menthol, the genus *Mentha* is known to be rich in phenolic compounds, including rosmarinic acid and caffeic acid, its major bioactive chemical constituents [9-11]. While few studies have already reported the bioactivities of fermented Mentha extract [12-14], a more detailed study focusing on bacterial strain-specific changes in metabolites and gene expression of metabolic enzymes involved in the fermentation of Mentha extract is yet to be explored. Thus, in this work, we report the increased bioactivity of the Mentha extract against LPS-stimulated RAW 264.7 cells by fermentation with strain-specific LAB, along with the changes in major phenolic acid concentrations, the detection of a newly produced bioactive metabolite, and the identification and overexpression of phenolic acid metabolism-related genes in *L. plantarum* SN13T during the fermentation*.*

## Materials and Methods

### Bacteria Culture and Fermentation Conditions

The lactic acid bacterial strains *Lactobacillus plantarum* SN13T and *Pediococcus pentosaceus* LP28, which have been isolated previously from plant sources—banana leaves and longan fruit *Euphoria longana*, respectively—were grown at 37 °C overnight in MRS broth (Merck, Germany). After cultivation, the bacterial cells were collected by centrifugation at 8000 × g for 10 min.

*Mentha arvensis* Linné var. *piperascens* Malinvaud (Lamiaceae), i.e., Mentha herb (5 g), purchased from Kojima Kampo Co., Ltd. (Osaka, Japan), was extracted by suspending and boiling in 100 ml of distilled water for 30 min. After cooling to room temperature, it was centrifuged at 5000 × g for 10 min and filtrated with a 0.22 µm membrane filter (Advantec Ltd., Japan) to obtain the aqueous extract of *Mentha arvensis* (MA). Then, the overnight bacterial cells (approximately 3 × 10^9^ colony forming units/ml) obtained by centrifugation were inoculated into the MA extract and incubated at 30 °C with shaking at 120 rpm. These conditions were chosen on the basis of previous literatures of plant-food fermentations where hydroxycinnamic acids (like rosmarinic acid and caffeic acid in Mentha extract) are abundant [15, 16]. After 24 h (h), the pH dropped to 4.4 in the fermented extracts (fMA-SN13T and fMA-LP28), and they were finally collected by centrifugation at 5000 × g for 10 min and subsequent filtration.

For the RT-qPCR experiments of bacterial phenolic acid metabolism genes, the overnight cells of *Lact. plantarum* SN13T were incubated with MRS, MRS supplemented with 1 mg/ml of rosmarinic acid (MRS + RA), or MA for 5 h or 24 h at 30 °C with shaking at 120 rpm.

### Cell Culture and Treatment

Murine macrophage-like cell line RAW 264.7 cells (RRID: CVCL_0493) were grown in DMEM medium supplemented with 10% FBS and 100 µg/ml of penicillin/streptomycin by incubation in a humidified 5% CO_2_ atmosphere at 37 °C, as described previously [7]. To stimulate the cells, the medium was exchanged with fresh DMEM medium with 0.5% FBS, and 1 µg/ml LPS was added in the presence or absence of the extracts (0.5% to 1% (*v/v*) final concentration) or standard solutions of rosmarinic acid (RA), caffeic acid (CA), or dihydrocaffeic acid (DHCA) at concentrations of 5 µg/ml to 60 µg/ml and incubated for 5 h or 24 h.

### Cell Viability Assay

Cell viability was determined using a CCK-8 kit (Dojindo, Japan) in accordance with the manufacturer’s instructions. Briefly, 100 μl of cells at a density of 1.8 × 10^5^ cells per well was incubated with LPS (1 µg/ml) in the presence or absence of the extracts or standard solutions for 24 h. CCK-8 solution (10 μl) was added to each well, and the cells were incubated for another 2 h. Then, the absorbance at 450 nm was measured. The percentage of viable cells was determined as a value relative to untreated cells.

### Measurement of Intracellular ROS Levels

The RAW 264.7 macrophage cells were treated with LPS in the presence or absence of the extracts or standard solutions for 24 h. After incubating the cells with 10 mM DCFH-DA for 30 min at 37 °C and washing twice with phosphate-buffered saline, the DCF fluorescence was measured at excitation and emission wavelengths of 485 and 530 nm, respectively [17]. The Relative Fluorescence Unit (RFU) thus obtained represented the intracellular ROS levels.

### Measurement of NO Production

The nitric oxide (NO) levels were determined by measuring the nitrite in the supernatant from the cell culture treated with LPS for 24 h. Equal volumes of the cell culture supernatants and the Griess reagent (1% sulfanilamide in 5% phosphoric acid and 0.2% naphthyl ethylenediamine dihydrochloride) were mixed. After 5 min at room temperature, the absorbance at 550 nm was measured. The NO concentration was calculated from the standard curve of sodium nitrite [18].

### Inflammatory Cytokine Determination

The cells were treated with LPS in the presence or absence of the extracts or standard solutions for 24 h. The concentrations of inflammatory cytokines IL-1β, IL-6, and TNF-α in the cell supernatants were determined by respective ELISA kits (BioLegend, USA), following the manufacturer’s protocols.

### RNA Extraction and qRT-PCR Analysis

RNA extraction and qRT-PCR were performed separately for samples treated with RAW 264.7 cells and bacterial cells grown in MRS and MA, to study the gene expression of LPS-stimulated inflammatory mediators and the gene expression of phenolic acid metabolism genes, respectively.

The total RNA from RAW 264.7 cells treated for 5 h with the extracts or standard solutions and *Lact. plantarum* SN13T cells grown in MRS, MRS-RA, and MA for 5 h or 24 h were isolated using NucleoSpin RNA plus (Macherey–Nagel GmbH and Co. KG, Germany). ReverTra Ace qPCR RT Master Mix with gDNA remover (Toyobo, Japan) was used for gDNA removal and reverse transcription in accordance with the manufacturer’s instruction manual. qRT-PCR was conducted on the CFX Maestro 2.3 real-time PCR system (Bio-Rad, USA) using the KAPA SYBR Fast qPCR Kit (Kapa Biosystems, USA). The qPCR was conducted under the following conditions: an initial 2 min at 95 °C, followed by 40 cycles of 5 s at 95 °C and 10 s at 60 °C. The relative transcriptional levels were normalized to housekeeping genes, i.e., *gapdh* for RAW 264.7 cells and *ldh* for bacterial cells. The gene expressions were analyzed using the ΔΔCT method. As given in Table 1, the primers used for qRT-PCR of LPS-induced inflammatory genes of RAW 264.7 cells were reported in a previous study [18], while the primers of SN13T genes were designed using Primer-BLAST online tool [19]. All qPCR assays amplified a single product, as determined by melting curve analysis and shown in Supplementary Figs. 1 and 2.

**Table 1:** Primers used in this study

**Primer**	**Forward** **(5′-** **>** **3′)**	**Reverse** **(5′-** **>** **3′)**
***gapdh*** ***iNOS*** ***sod 2*** ***il-1β*** ***il-6*** ***tnf-α*** ***ldh*** ***hcrA*** ***hcrB*** ***hcrR*** ***hcrC*** ***ceh***	GACATCATACTTGGCAGG GGTGTTGAAGGGGTAGCTGA GTGACTTTGGGTCTTTTGA ATGGCAACTGTTCCTGAACTCAACT ACAGGTCTGTTGGGAGTGGTATC AGCCCCCAGTCTGTATCCTT GCCGACGAAGGGGTTAAGAA GGCGCTTCTAAGATTTGCCG CTTAAGCGTGCGGAAATCGG GCCGTTGTTTTTACCCGCTT GTACTTGGTGCCCTTCGTCA GCCCCACTTCTCCATTTCCA	CTCGTGGAGTCTACTGGT ATCATGGACCACCACACAGC GCTAACATTCTCCCAGTT CAGGACAGGTATAGATTCTTTCCTTT CTCTCTGCAAGAGACTTCCATCC CTCCCTTTGCAGAACTCAGG GTAGGTATCGAGGGCAGCAC GGGGTGATTTTTGCAACCCC GTGATGATTGTCGGCGCTTC GTGGACGCTGTCTGGAAGAA CAGGTGTTTCAGACGCTTGC TGCAGGAGCTTGAACACGAT

### Identification and Determination of Metabolites in Unfermented and Fermented Mentha Extract

HPLC analyses were performed to compare the constituents of unfermented and fermented Mentha extracts according to a previous study with modifications [7]. Aliquots (2.5 µl) of the MA, fMA-SN13T, and fMA-LP28 extracts were applied to HPLC (JASCO system; JASCO Corporation) with a YMC-Pack ODS-AQ (150 × 4.6 mm, 5 µm, 12 nm) column (YMC, Japan). The column was equilibrated with water containing 0.1% trifluoracetic acid, and gradient elution was performed with 0% to 10%, 10 to 40%, and finally 40 to 60% acetonitrile over 20 min, 30 min, and 10 min sequentially at a flow rate of 1 ml/min. The elution profiles were monitored at absorbances of 320 nm and 280 nm. The chromatogram of the MA was compared with those of the fermented extracts, fMA-SN13T and fMA-LP28. Rosmarinic acid and caffeic acid in all of the extracts were identified using the respective analytical standards, and their concentrations were determined by standard curves at the absorbance of 320 nm. At 280 nm, a newly produced compound was detected in fMA-SN13T. The compound was purified from fMA-SN13T by ethyl acetate extraction, followed by preparative TLC using a mobile phase of methanol: chloroform: toluene at 5:4:1, and followed by HPLC.

The purified compound was identified using a combination of GC–MS as well as ^1^H-NMR and ^13^C-NMR spectra. The MS spectra were captured on a Thermo Fisher Scientific LTQ Orbitrap XL. GC–MS was performed on a JMS-T100GCV AccuTOF GCv4G (JEOL Ltd.) with an HP-5MS capillary column (0.25 mm × 0.25 µm × 30 m). The parameters for EI mode were an ion-source temperature of 250 °C, an electron energy of 70 eV, and a filament current of 300 µA. ^1^H-NMR and ^13^C-NMR spectra were captured on a JEOL JNM-LA500 spectrometer at 600 MHz. Finally, the identified compound was confirmed by HPLC using the analytical standard.

### Sequence Data Analyses

Homology searches were performed on the whole genome sequences of *Lact. plantarum* SN13T and *Ped. pentosaceus* LP28 using the software in silico Molecular Cloning (R) Genomics Edition version 6.0.30D and the BLAST algorithm on the National Center for Biotechnology Information server (https://blast.ncbi.nlm.nih.gov/Blast.cgi) [20], respectively. Multiple alignments were made using the Clustal Omega program (https://ebi.ac.uk/Tools/msa/clustalo/) on the EBI site after the retrieval of sequences from the GenBank database [21].

### Statistical Analyses

All data were presented as the means and SD of triplicates. The significance of differences was determined via ANOVA, followed by a post hoc Tukey test, and differences with *p* < 0.05 were considered statistically significant.

## Results

### Increased Bioactivity of MA Extract by Fermentation with *Lact. plantarum* SN13T

**Fig. 1:**
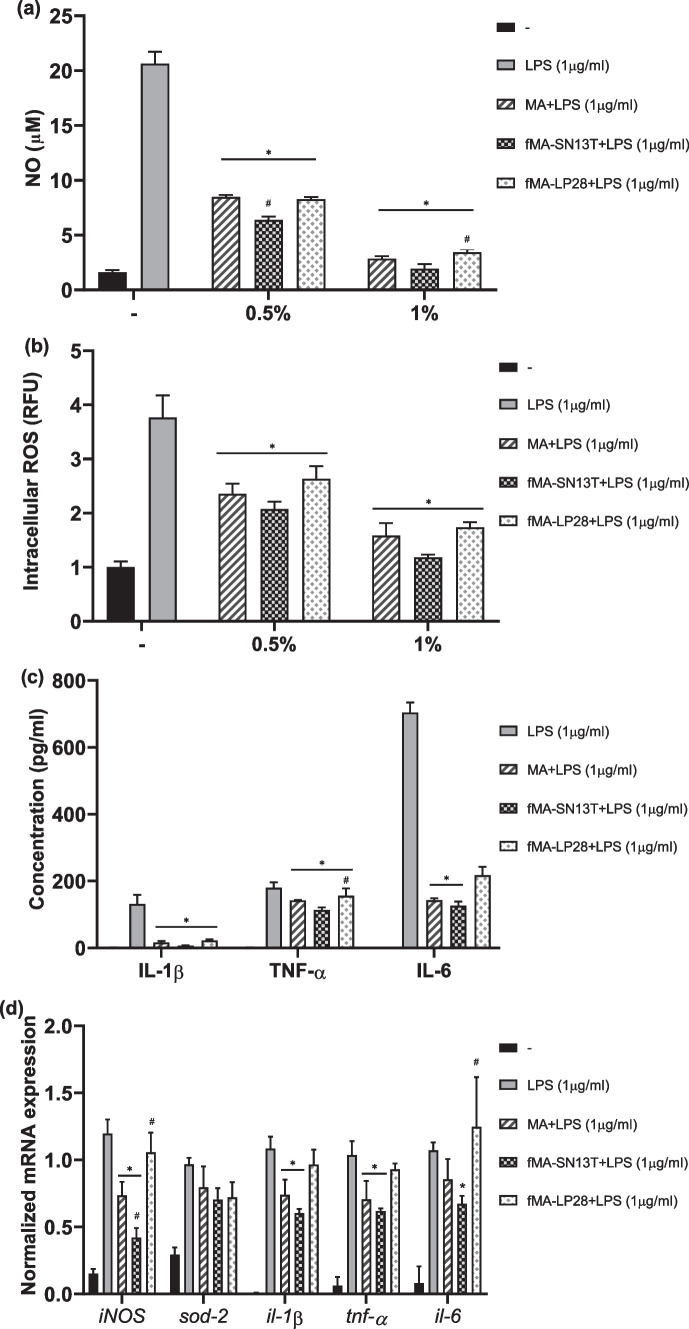
Increased bioactivity of MA extract by fermentation with strain-specific *Lactobacillus* strains. **a** Nitric oxide level (µM) in LPS-stimulated RAW 264.7 cells treated with the extract MA, fMA-SN13T, or fMA-LP28, expressed in comparison with the control. **b** Intracellular ROS levels (RFU) in LPS-stimulated RAW 264.7 cells treated with MA, fMA-SN13T, or fMA-LP28, expressed in comparison with the control. **c** Cytokine (IL-1β, TNF-α, and IL-6) levels (pg/ml) in LPS-stimulated RAW 264.7 cells treated with MA, fMA-SN13T, or fMA-LP28, expressed in comparison with the control. **d** Relative mRNA expressions of *iNOS*, *sod-2*, *il-1β*, *tnf-α*, and *il-6* in LPS-stimulated RAW 264.7 cells treated with MA, fMA-SN13T, or fMA-LP28. All data are expressed as the mean value of triplicate experiments. Error bars represent ± standard deviation. ^*^p < 0.05 versus LPS and ^#^*p* < 0.05 versus MA-treated cells

When RAW 264.7 cells were stimulated with LPS (1 µg/ml) for 24 h, the levels of inflammatory mediators NO, ROS, IL-1β, IL-6, and TNF-α in cell supernatants were found to be significantly more increased than those in untreated cells, as seen in Fig. 1a–c. At a 0.5% or 1% concentration, all of the extracts displayed cell viability similar to that of control cells. Figure 1a shows that, in the presence of MA or fermented MA at 0.5% or 1% final concentrations, the LPS-induced NO levels were significantly decreased. At 0.5%, significant decreases in NO as compared to the unfermented MA were observed in fMA-SN13T but not in fMA-LP28. Similar results are seen in Fig. 1b, where all of the extracts—MA, fMA-SN13T, and fMA-LP28—could significantly decrease the LPS-induced intracellular ROS levels at both concentrations. The highest decrease was shown in fMA-SN13T, although it was not significantly higher than in other extracts. Meanwhile, Fig. 1c depicts significant decreases in the levels of LPS-induced cytokines IL-1β, IL-6, and TNF-α after treatment with MA or fermented MA at a 1% concentration. Here, as well, fMA-SN13T was found to be the most bioactive of the extracts.

To confirm the bioactivity of MA and fermented MA extracts, the relative gene expression levels of *iNOS*, *sod-2*, *il-1β*, *il-6*, and *tnf-α* in RAW 264.7 cells stimulated with LPS for 5 h were determined in the presence of the extracts MA, fMA-SN13T, and fMA-LP28 at a 0.5% concentration, as shown in Fig. 1d. While the relative gene expression of inflammation-related NO-producing enzyme *iNOS* was significantly downregulated in the presence of MA and fMA-SN13T, it was notable that its expression in the presence of fMA-SN13T was seen to be significantly reduced even as compared to that of MA. However, the treatment of fMA-LP28 could not significantly reduce the *iNOS* mRNA expression as compared to that of LPS-treated cells. Consistent with the results of the previous experiment, the fermented or unfermented MA could decrease the LPS-induced gene expressions of inflammatory cytokines *il-1β*, *il-6*, and *tnf-α* and antioxidant enzyme *sod-2*. In these cases, the fermented extract, fMA-SN13T, had higher activity than MA, while fMA-LP28 extract demonstrated the least activity.

### Metabolism of Rosmarinic Acid (RA) in MA by *Lact. plantarum* SN13T

**Fig. 2:**
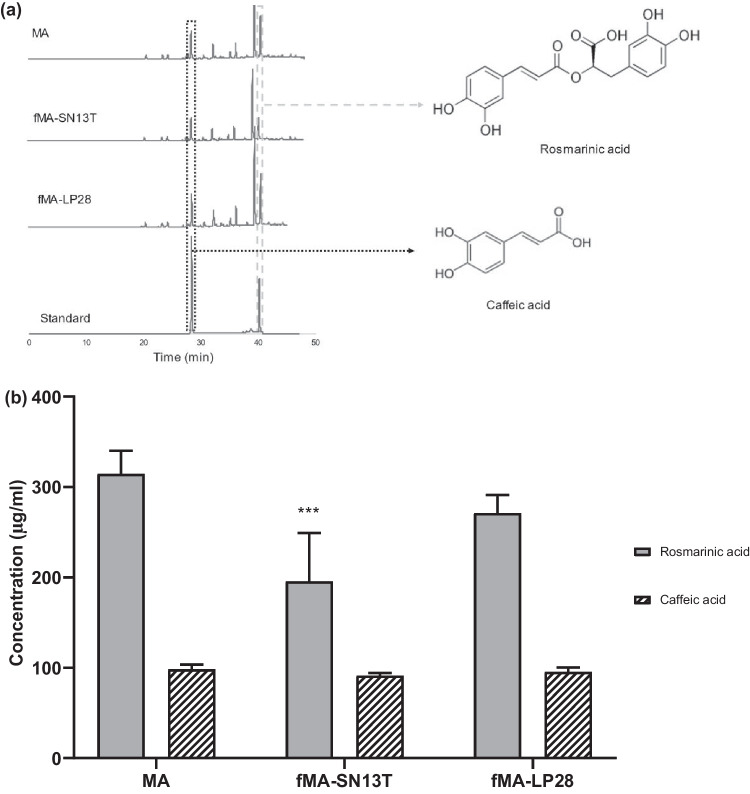
**a** HPLC chromatograms of MA, fMA-SN13T, and fMA-LP28 extracts and the analytical standards of rosmarinic acid and caffeic acid at the absorbance of 320 nm. The peaks enclosed in boxes with gray dashes and black dots represent rosmarinic acid and caffeic acid, respectively. **b** Concentrations (µg/ml) of rosmarinic acid and caffeic acid measured in MA, fMA-SN13, and fMA-LP28 extracts as determined from HPLC standard curves. Data are expressed as the mean value of triplicate experiments. Error bars represent ± standard deviation. ^***^p < 0.01 versus MA

We performed HPLC analyses of the extracts MA, fMA-SN13T, and fMA-LP28 to compare the chemical constituents in the MA extract after fermentation. In Fig. 2a, the HPLC chromatograms of the extracts detected at the absorbance of 320 nm show peaks of rosmarinic acid and caffeic acid at retention times of 39.52 min and 27.18 min, respectively. It was observed that the concentration of rosmarinic acid significantly decreased in fMA-SN13T as compared to that in unfermented MA—from 314.45 µg/ml to 192 µg/ml—while only an insignificant decrease in the fMA-LP28 extract, and no significant change in the caffeic acid concentration was measured among the three extracts as shown in Fig. 2b.

### Identification of a Newly Produced Compound in the Fermented Extract

**Fig. 3:**
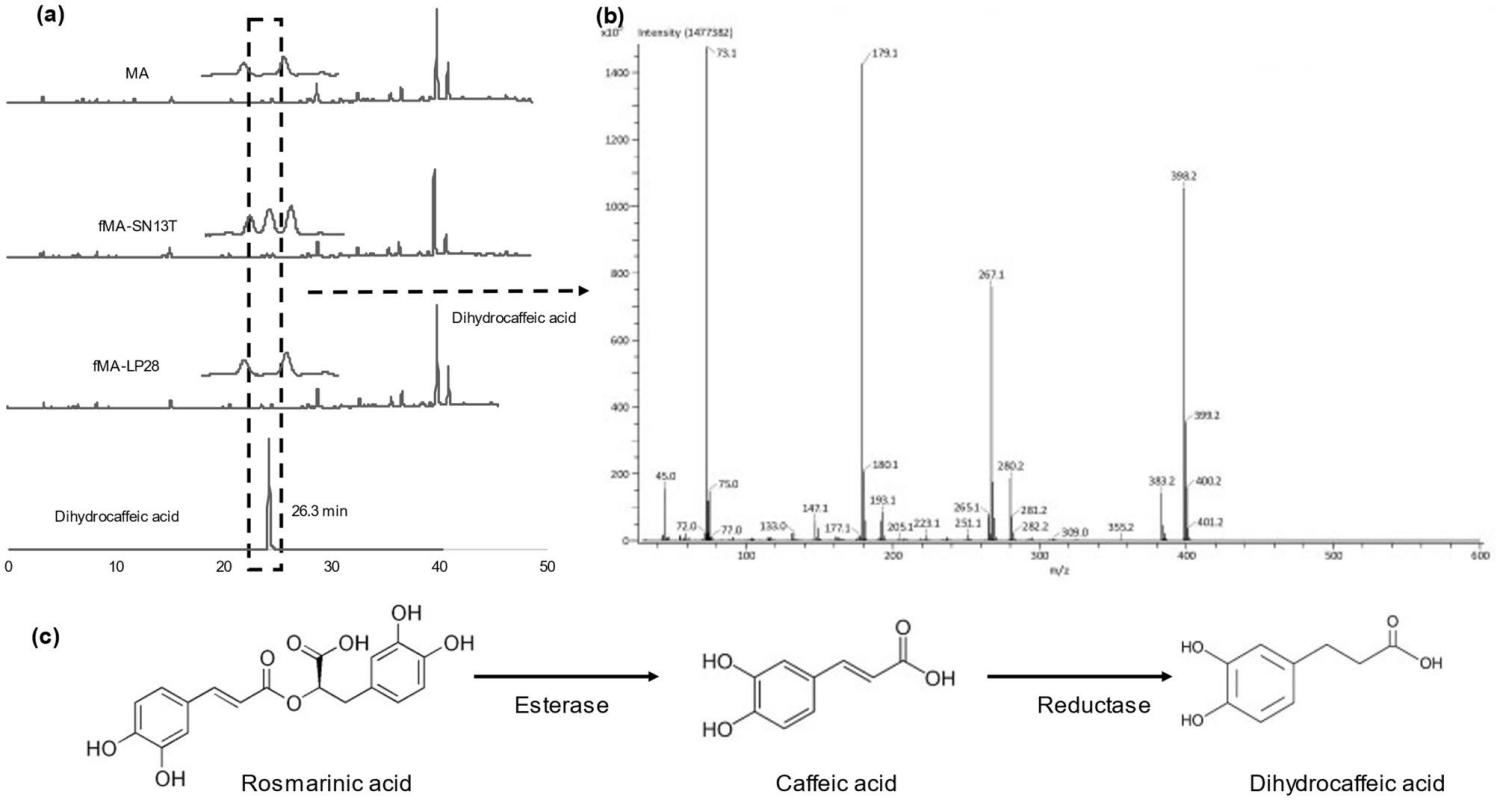
**a** HPLC chromatograms of MA, fMA-SN13T, and fMA-LP28 extracts and analytical standards of dihydrocaffeic acid at the absorbance of 280 nm. **b** GC–MS spectra (EI mode) of purified dihydrocaffeic acid derivatized with TMS. **c** Possible metabolic pathway of dihydrocaffeic acid from rosmarinic acid via caffeic acid by the actions of bacterial esterase and reductase

On further analyses of the HPLC chromatograms of the extracts MA, fMA-SN13T, and fMA-LP28 at the absorbance of 280 nm, we detected a newly produced peak at the retention time of 23.6 min in the fMA-SN13T extract. However, it was undetectable in both unfermented MA and fMA-LP28, as shown in Fig. 3a. The accumulation of this metabolite in fMA-SN13T could be responsible for the higher bioactivity than in the other extracts. Therefore, we purified this compound from the extract fMA-SN13T sequentially by organic solvent extract, preparative TLC, and HPLC. For the identification, this purified compound was analyzed for GC–MS, ^1^H-NMR, and ^13^C-NMR spectra, as shown in Figs. 3b, S3a, and S3b, respectively. The number of molecular ions of the underivatized compounds detected as [M–H]^−^ in the negative ion ESI–MS spectra was determined to be 182, with the molecular formula C_9_H_10_O_4_. The GC–MS data showed three TMS groups on the derivatized sample with a calculated M^+^ of 398 and fragment ions (m/z) of 383, 282, 267, and 179. The δ_C_ (chemical shifts in ppm) in ^13^C-NMR spectra were observed at 179.957, 146.203, 144.604, 133.746, 120.447, 116.339, 37.156, and 31.469. Taken together, the NMR data and MW data were consistent with previous reports of the 2,3-dihydro derivative of caffeic acid [22]. Finally, after confirming the purified peak to be 3-(3′,4′-dihydroxyphenyl) propanoic acid or dihydrocaffeic acid (DHCA) by comparing the HPLC chromatogram of its analytical standard, we determined its concentration produced in fMA-SN13T to be 71.78 µg/ml. The possible metabolic pathway, by which the excess caffeic acid in the MA extract fermented with *Lact. plantarum* SN13T formed by the hydrolysis of rosmarinic acid via bacterial esterases is further reduced into dihydrocaffeic acid via reductases, is depicted in Fig. 3c.

### Dihydrocaffeic Acid is a More Potent NO Inhibitor Than Caffeic Acid and Rosmarinic Acid

**Fig. 4:**
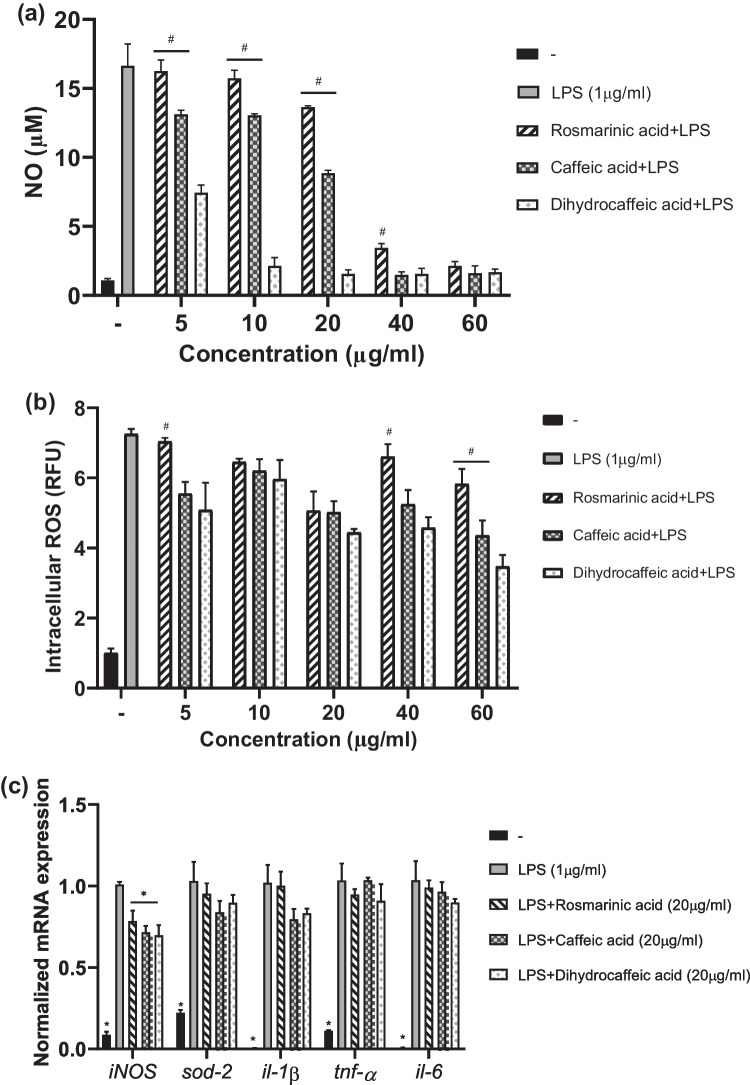
**a** Effect of rosmarinic acid (RA), caffeic acid (CA), and dihydrocaffeic acid (DHCA) on NO levels (µM) produced by LPS-induced RAW 264.7 cells at concentrations of 5 µg/ml, 10 µg/ml, 20 µg/ml, 40 µg/ml, and 60 µg/ml. **b** Effects of RA, CA, and DHCA on intracellular ROS levels (RFU) produced by LPS-induced RAW 264.7 cells at concentrations of 5 µg/ml, 10 µg/ml, 20 µg/ml, 40 µg/ml, and 60 µg/ml. **c** Effect of RA, CA, and DHCA on relative mRNA expressions of *iNOS*, *sod-2*, *il-1β*, *tnf-α*, and *il-6* in LPS-induced RAW 264.7 cells at a concentration of 20 µg/ml. Data are expressed as the mean value of triplicate experiments. Error bars represent ± standard deviation. ^*^p < 0.05 versus LPS and ^#^*p* < 0.05 versus DHCA-treated cells

We compared the activities of RA, CA, and DHCA in various concentrations in our experimental model of LPS-induced RAW 264.7 cells. All three had decreased cell viability above a concentration of 80 µg/ml; thus, we determined the NO and intracellular ROS levels of LPS-induced cells when treated with concentrations of 5 µg/ml, 10 µg/ml, 20 µg/ml, 40 µg/ml, and 60 µg/ml. As seen in Fig. 4a, at the highest concentration of 60 µg/ml, all RA, CA, and DHCA could significantly lower the LPS-induced NO in RAW 264.7 cells. At lower concentrations, from 5 µg/ml to 20 µg/ml, DHCA markedly decreased the NO levels as compared to both CA and RA; i.e., DHCA was the most potent inhibitor of LPS-induced NO. Similarly, Fig. 4b shows that CA and DHCA decreased the LPS-induced intracellular ROS significantly as compared to RA at concentrations of 5 µg/ml, 40 µg/ml, and 60 µg/ml. In addition, all RA, CA, and DHCA at a concentration of 20 µg/ml significantly decreased the mRNA expression of *iNOS* as compared to the LPS-only-treated cells, but any significant differences between the sample groups were not observed in the case of the antioxidant enzyme *sod-2* gene and proinflammatory cytokine *il-1β*, *tnf-α*, and *il-6* genes, as shown in Fig. 4c.

### Identification of Phenolic Acid Metabolism-Related Genes in *Lact. plantarum* SN13T

On analysis of the complete genome sequences, AP019815.1 and NZ_DF970691.1 of *Lact. plantarum* SN13T and *Ped. pentosaceus* LP28, respectively, for annotated cinnamoyl ester hydrolase (*ceh*), none was found in strain LP28, while a putative enzyme with 114 amino acids was found in SN13T (*SN13T_*1651), showing 35% similarity, as given in Table 2, and aligned with *ceh* homolog *Lj0536* in *Lact. johnsonii* N6.2 in Fig. S4a. As shown in Table 2, SN13T harbored the complete *hcr* operon, consisting of a Lys-R type transcriptional regulator, NADPH-dependent FMN reductase family protein, NADPH-dependent FMN reductase, and a hypothetical protein encoded by *SN13T_0395*, *SN13T_0394*, *SN13T_0393*, and *SN13T_0392* that exhibited only 86%, 94%, 90%, and 85% amino acid identity to *hcrR*, *hcrA*, *hcrB*, and *hcrC* of *Lact. plantarum* WCFS1, respectively (see Figure S4b-e, respectively).

**Table 2:** In silico identification of putative phenolic acid esterase and reductase in *Lact. plantarum* SN13T

**Putative** **enzyme**	**Accession** **no.**	**Protein** **length** **(aa)**	**Percent** **identity** **(%)**	**Query protein**
*ceh* (Cinnamoyl ester hydrolase)	*SN13T_1651*; BBM21616.1	114	35	*Lact. johnsonii* N6.2 WP_004898050, 249 aa
*hcrR* (transcription regulator, Lys-R family)	*SN13T_0395*; BBM20405.1	315	86	*Lact. plantarum* WCFS1 YP_004889724.1, 315 aa
*hcrA* (NADPH-dependent FMN reductase family protein)	*SN13T_0394*; BBM20404.1	204	94	*Lact. plantarum* WCFS1 YP_004889725.1, 204 aa
*hcrB* (NADPH-dependent FMN reductase)	*SN13T_0393*; BBM20403.1	812	90	*Lact. plantarum* WCFS1 YP_004889726.1, 812 aa
*hcrC* (Hypothetical protein)	*SN13T_0392*; BBM20402.1	186	85	*Lact. plantarum* WCFS1 YP_004889727.1, 186 aa

### Overexpression of Phenolic Acid Metabolism-Related Genes in *Lact. plantarum* SN13T During MA Fermentation

**Fig. 5:**
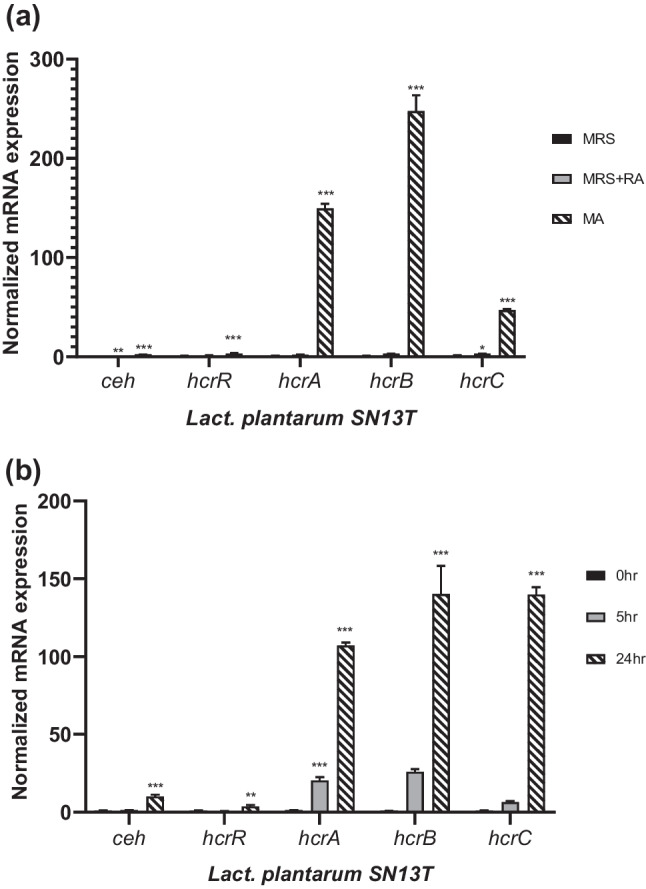
**a** Relative mRNA expressions of phenolic metabolism-encoding genes *ceh*, *hcrR*, *hcrA*, *hcrB*, and *hcrC*, when *Lact. plantarum* SN13T was grown in MRS, MRS with rosmarinic acid (1 µg/ml), and MA for 5 h. **b** Relative mRNA expressions of phenolic metabolism–encoding genes *ceh*, *hcrR*, *hcrA*, *hcrB*, and *hcrC*, when *Lact. plantarum* SN13T was grown in MA for 0 h, 5 h, or 24 h. Data are expressed as the mean value of triplicate experiments. Error bars represent ± standard deviation. ^*^p < 0.05, ^**^p < 0.01, and ^***^p < 0.001 versus MRS or 0 h

After the identification of phenolic acid metabolism-related genes in *Lact. plantarum* strain SN13T, we speculated that these genes were overexpressed during the fermentation of the plant extract MA. Hence, we compared the expressions of genes *ceh*, *hcrR*, *hcrA*, *hcrB*, and *hcrC* when these strains were grown in MRS, MRS supplemented with RA at 1 µg/ml, and MA for 5 h. As shown in Fig. 5a, the esterase gene *ceh* was significantly upregulated in the presence of RA, as well as the MA extract, as compared with MRS broth. In MA, the relative expression of *hcrB* reached higher than 250-fold as compared to MRS, along with the significant overexpression of the remaining *hcr* operon. Additionally, it was found that the esterase gene *ceh* and all genes *hcrRABC* of reductase operon were increasingly upregulated at 5 h and 24 h of growth, as shown in Fig. 5b, which supports that phenolic acid-metabolism genes of *Lact. plantarum* SN13T are time-dependently overexpressed, producing DHCA in the MA extract during the 24 h fermentation.

## Discussion

Our results demonstrated that fermentation with *Lact. plantarum* SN13T could enhance the potency of the MA extract, while fermentation with *Ped. pentosaceus* LP28 did not show significantly better bioactive properties in the in vitro experimental model of LPS-stimulated RAW 264.7 murine macrophages. An anti-inflammatory drug, dexamethasone at 0.5 µg/ml, also significantly reduced NO concentration and *iNOS* expression in the same in-vitro model in our previous unpublished study, which is comparable to the bioactivity of unfermented or SN13T fermented Mentha extracts (see Supplemental Figure S5). The LPS-induced RAW 264.7 macrophage model was also used to study the anti-inflammatory effect of the stem extract of *Alternanthera sessilis*, where its potential to suppress the proinflammatory mediators and cytokines was compared to dexamethasone [23]. In our previous study, fermentation with *Lact. brevis* 174A strain enhanced the antioxidant and anti-inflammatory activities of the medicinal herb extract *Paeonia Radix* Alba by increasing the total phenolic content (TPC) and producing the gallic acid metabolite, pyrogallol [7]. Numerous other reports have also shown that changes in the phenolic content are associated with simultaneous changes in antioxidant activities after *Lactobacillus* fermentation of various food matrices, due to the enzymatic actions of any of the commonly inherited enzymes of *Lactobacillus* species, such as glucosidase, esterase, phenolic acid decarboxylase, phenolic acid reductase, or tannase [24-29]. In addition, it has been well reported that the ability of LAB to improve the bioavailability and bioactivity of phytochemicals is associated with species- or strain-specific metabolic features [3]. The strain *Lact. plantarum* SN13T has been previously found to produce IL-8-inhibiting metabolites like catechol and secotanapartholide C in the medicinal herb extract of *Artemisia princeps* Pampanini. It was shown that this strain could grow vigorously in different herbal extracts, as it harbors a β-glucosidase enzyme encoding 11 open reading frames (ORFs) [6]. Thus, we speculated that, when grown in the Mentha herb, *Lact. plantarum* SN13T also produces bioactive metabolites that potentiate its bioactivity as compared to other strains.

In the genus *Mentha*, rosmarinic acid and caffeic acid are reported to be the most important bioactive phenolic acid metabolites [30]. In fact, rosmarinic acid is one of the marker compounds used to evaluate the quality of *M. arvensis* L. [31]. Thus, the significant decrease in RA concentration after SN13T fermentation of MA was a notable observation as it could be co-related with the significantly increased bioactivity of fMA-SN13T extract. However, it has been reported that *M. arvensis* L. extract fermented with a combination of *Lact. rhamnosus*, *E. faecium*, and *Lact. acidophilus* showed an increase in the concentration of rosmarinic acid that could suppress MDA, NO, and corticosterone; improve body weight; decrease daily food intake and duodenum histology; and increase serotonin and β-endorphin levels in immobilization-induced stressed rats [12]. In another study, such fermented *M. arvensis* extract administration also provided neuroprotection against transient global cerebral ischemia in gerbils and SH-SH5Y cells by downregulating MAPK signaling [13]. In addition, *M. piperita* fermented with *Bacillus subtilis* suppressed PMA-induced ROS, ERK, and MUC5AC mRNA protein expression in lung epithelial cells [14]. Nonetheless, these previous reports confirm the functional aspects of the strain-specific fermentation of *Mentha* herb extracts, but without a clear understanding of the metabolite profiling of such fermented extracts.

Meanwhile, there have been several reports suggesting that rosmarinic acid can be readily degraded into caffeic acid and derivatives by gut microbiota in vitro as well as after oral intake in mice and humans [32, 33]. *Lact. johnsonii* was reported to metabolize RA from rosemary extract with cinnamoyl esterases into caffeic acid and 3,4-dihyroxyphenyllactic acid in vitro and in a gastrointestinal model [34]. The microbial catabolism of RA was also confirmed in thyme phenol-enriched olive oil, yielding hydroxyphenylpropionic acid as the main metabolite via CA [35]. RA was also reported to be degraded into conjugated forms of caffeic acid, ferulic acid, and m-coumaric acid following intake of *Perilla frutescens* [36]. While the immediate metabolite of RA has been consistently identified as CA in several reports [30, 34, 35, 37], CA is known to be either reduced into dihydrocaffeic acid or decarboxylated into vinyl catechol via microbial phenolic acid reductase or decarboxylase, respectively [38, 39]. Other studies have reported the final metabolite of CA to be 3-hydroxyphenylpropionic acid (3-HPP) or 4-ethylcatechol via further degradation [35, 40, 41]. Since there was no significant change in the CA concentration among the three extracts in our study, this led us to hypothesize that the CA produced from rosmarinic acid was further degraded into other bioactive compounds in fMA-SN13T. In our results, only DHCA was detected as a phenolic acid metabolite in the SN13T-fermented MA. Strain-specific metabolism of CA during in vitro *Lactobacillus* fermentation of phenolic-rich plant foods such as cherry juice, broccoli puree, and elderberry juice also produced DHCA through phenolic acid decarboxylases and reductases [42, 43].

To explore the possibility of the increased bioactivity of the fMA-SN13T extract as compared to unfermented MA and fMA-LP28 extracts to be associated with the concentration of DHCA, we studied its bioactivity with respect to its metabolic precursors, RA and CA. RA, CA, and caffeoyl derivatives have been reported to have in vitro antioxidant activity to scavenge superoxide and hydroxyl radicals and a potent anti-inflammatory activity resulting from decreased arachidonate formation in previous studies [44, 45]. Our results suggest that the bioactivity of the phenolic acid metabolites against LPS-induced RAW 264.7 cells could be in the increasing order of RA, CA, and DHCA. Although DHCA is not one of the most commonly found hydroxycinnamic acids, its precursors, such as feruloyl podospermic acid, catechins, procyanidins, and caffeic acid, are found in different food sources [46]. It has been determined to be one of the major phenolic acids found in human fecal water, blood, and urine as a metabolite after the intake of various polyphenols present in food, beverages, and medicinal plants or extracts—such as coffee, artichoke leaf extracts, and chocolate—and in rat urine after the ingestion of polyphenol-rich wine extract [47-50]. Similar to our results, other studies have reported DHCA to be the most potent anti-DPPH (2,2-diphenyl-1-picrylhydrazyl radical) compound among CA, DHCA, and their corresponding n-alkyl esters [51]. DHCA, along with the related colonic metabolite dihydroferulic acid, was found to be more effective inhibitors of in vitro platelet activation than their phenolic precursors present in green coffee bean extract and yerba mate extract via P-selectin expression, suggesting an increase in efficacy with the metabolism of phenolic compounds [52]. DHCA can also function as an intracellular antioxidant in human EA.hy926 epithelial cells by increasing *eNOS* activity [53]. It reduces the cytotoxicity and pro-inflammatory cytokine IL-6 and IL-8 production in UV-irradiated HaCaT keratinocyte cells, resulting from the combined effect of direct radical scavenging of the ROS or reinforcement of the antioxidant potential of the keratinocytes, and a direct interference with the cytokine signaling pathway [46]. Recently, a high-throughput screening identified DHCA as being one of the two phytochemicals effective in promoting resilience against stress via reducing IL-6 production by inhibiting DNA methylation, which modulated the brain synaptic plasticity and peripheral inflammation, making it a strong candidate for treating stress disorders and depression, either alone or in combination with currently available antidepressants [54].

*Lactobacillaceae* are known to possess a broad spectrum of enzymes—such as esterases, reductases, and decarboxylases—for the biotransformation of bioactive dietary phenolic compounds such as hydroxycinnamic acids and hydroxybenzoic acids [39]. Several *Lactobacillus* strains have been characterized for their cinnamoyl esterase activity; however, such esterases—Lj0536 and Lj1228, reported to have substrate specificity for RA—have only been characterized for *Lact. johnsonii* N6.2 [37]. The *ceh* gene annotated in the whole genome sequence of *Lact. plantarum* SN13T encodes an alpha/beta fold hydrolase family enzyme, displaying no significant similarities with previously reported esterases *Lj1228*, *HceP*, *Lp_0796*, and *Est_1092* from *Lact. johnsonii* N6.2, *Lact. plantarum* TMW1.46, *Lact. plantarum* WCFS1, and *Lact. plantarum* DSM 1055, respectively [39]. These findings reinforce that the characterization of the substrate specificity of hydroxycinnamic acid esterases is limited to the use of a few model compounds and does not reflect the diversity of phenolic acid esters in plants and even in esterases in *Lact. plantarum* species.

On the other hand, the gene cluster involved in hydroxycinnamate reduction, *hcrAB*, was identified and characterized recently in *Lact. plantarum* WCFS1 [15]. The inducible reductase activity was not widely present among lactic acid bacteria and was reportedly 100 times lower than decarboxylase activity [55]. The presence of a functional *hcrB* gene was reported to be a minimum criterion for hydroxycinnamate reductase activity in *Lact. plantarum* [16]. Unlike in *Ped. pentosaceus* LP 28, putative phenolic acid reductases were identified in the complete genome of *Lact. plantarum* strains SN13T homologous to *hcrB* in *Lact. plantarum* WCFS1, along with other proteins that were encoded in the proposed operon, including *hcrR*, *hcrA*, and *hcrC*. Homologs of *hcrB* were also identified in *Lact. rossiae (Par1)* and *Lact. fermentum (hcrF)* having 25% and 63% amino acid identity, respectively [16]. In the same study, phenolic acid reductases were not identified in any of the *Pediococcus* strains, which is consistent with our report. It has been suggested that heterofermentative lactic acid bacteria use hydroxycinnamic acids as external acceptors of electrons to gain additional metabolic energy to combat the stressful conditions generated by phenolic acids [38]. In turn, this metabolic adaptive feature might be responsible for increasing the bioactivity and bioavailability of dietary phenolic acids.

The differential relative gene expressions of *hcrR*, *hcrA*, *hcrB*, and *hcrC* have been previously reported for *Lact. plantarum* WCFS1 when exposed to different hydroxycinnamic acids, such as p-coumaric acid, m-coumaric acid, o-coumaric acid, ferulic acid, caffeic acid, and sinapic acid [15]. These genes were also induced in the presence of a nonhydroxy-derived cinnamic acid, possibly, due to structural similarity, but not in the presence of hydroxybenzoic acids such as gallic acid [15]. Although slight similarity of the amino acid in the reductase enzymes was observed, the patterns of relative expression of *hcrR*, *hcrA*, *hcrB*, and *hcrC* genes by *Lact. plantarum* SN13T in the presence of RA or MA were found to be different from those of *Lact. plantarum* WCFS1 when exposed to caffeic acid [15]. The biotransformation of phenolic compounds by lactic acid bacteria does not always correlate with the presence or absence of enzymes and the metabolic activity in laboratory media [39], which may be associated with differential enzyme gene expressions in the presence of complex substrates. Such strain-specific expressions of genes encoding phenolic metabolism and their substrate-based differential regulation were also reported in olive extract, millet, and sorghum fermentations [56, 57]. Hence, our results support the notion that strain-specific enzymes metabolizing phenolic acids, the differential expression of genes encoding-related enzymes, and unlimited variation in composition of individual fermentation sources—foods, medicinal extracts, or media—ultimately affect the behavior of fermenting microbiota and their functional aspects.

To summarize, the fermentation of the herb Mentha extract with *Lact. plantarum* SN13T increases its antioxidant and anti-inflammatory bioactivity against LPS-induced macrophage cells. This effect was associated with the metabolism of RA into possibly potent metabolite DHCA via the overexpression of microbial strain-specific phenolic metabolism genes, i.e., esterase *ceh* and reductases *hcrR*, *hcrA*, *hcrB*, and *hcrC* in Mentha extract fermentation*.* Hence, we conclude that fermenting medicinal herbal extracts with specific plant-derived LAB strains could be a significant technique for enhancing their therapeutic potential.

### Supplementary Information

Below is the link to the electronic supplementary material.

Supplementary file1 (DOCX 1458 KB)

## Data Availability

All datasets generated for this study are included in the article/Supplementary Material.
