# Supplementary material for: Role of Phenolic Acid Metabolism in Enhancing Bioactivity of Mentha Extract Fermented with Plant-Derived Lactobacillus plantarum SN13T
Source: Probiotics Antimicrob Proteins. 2023 Jun 6;16(3):1052–64. doi: 10.1007/s12602-023-10103-4 (PMC11126511; doi:10.1007/s12602-023-10103-4)
Supplement: Supplementary file 1 — Supplementary file1 (DOCX 1458 KB) [file 12602_2023_10103_MOESM1_ESM.docx]

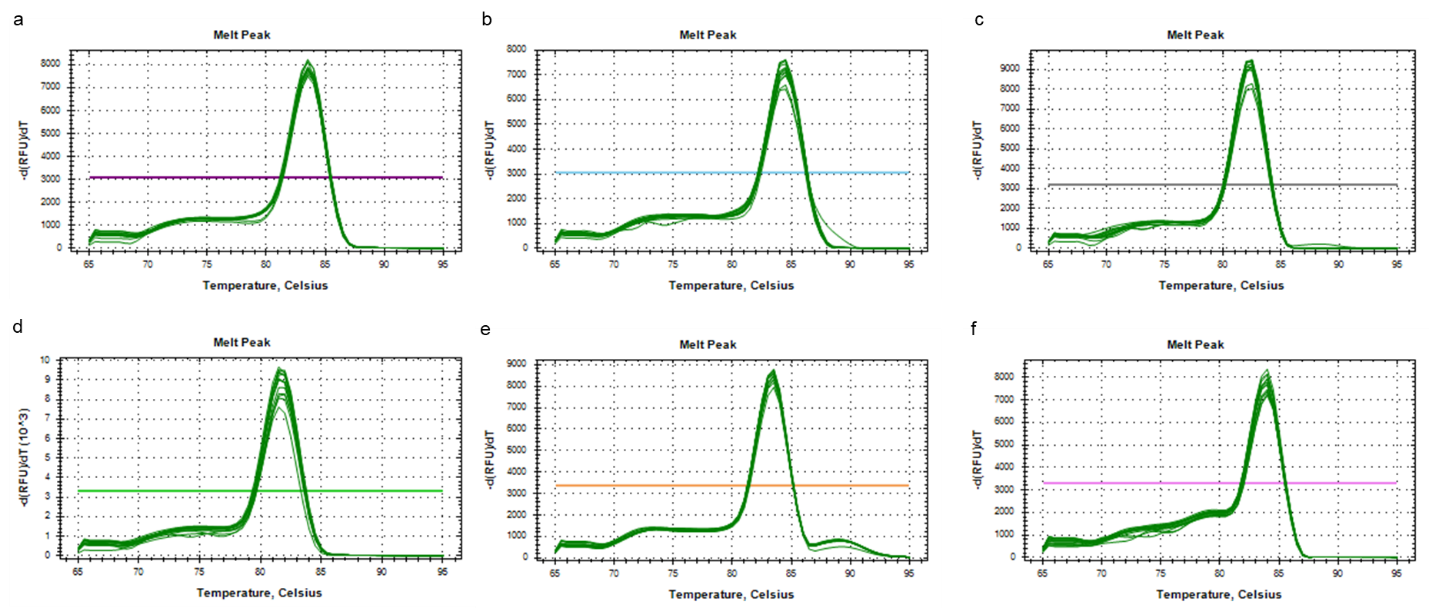


**S1**

**Figure S1** The melt curve of inflammatory genes (a) *gapdh*; (b) *iNOS*; (c) *tnf-α*; (d) *il-6*; (e) *il-1β*; and (f) *sod-2*, during qRT-PCR of LPS-stimulated RAW 264.7 cells.

**S2**


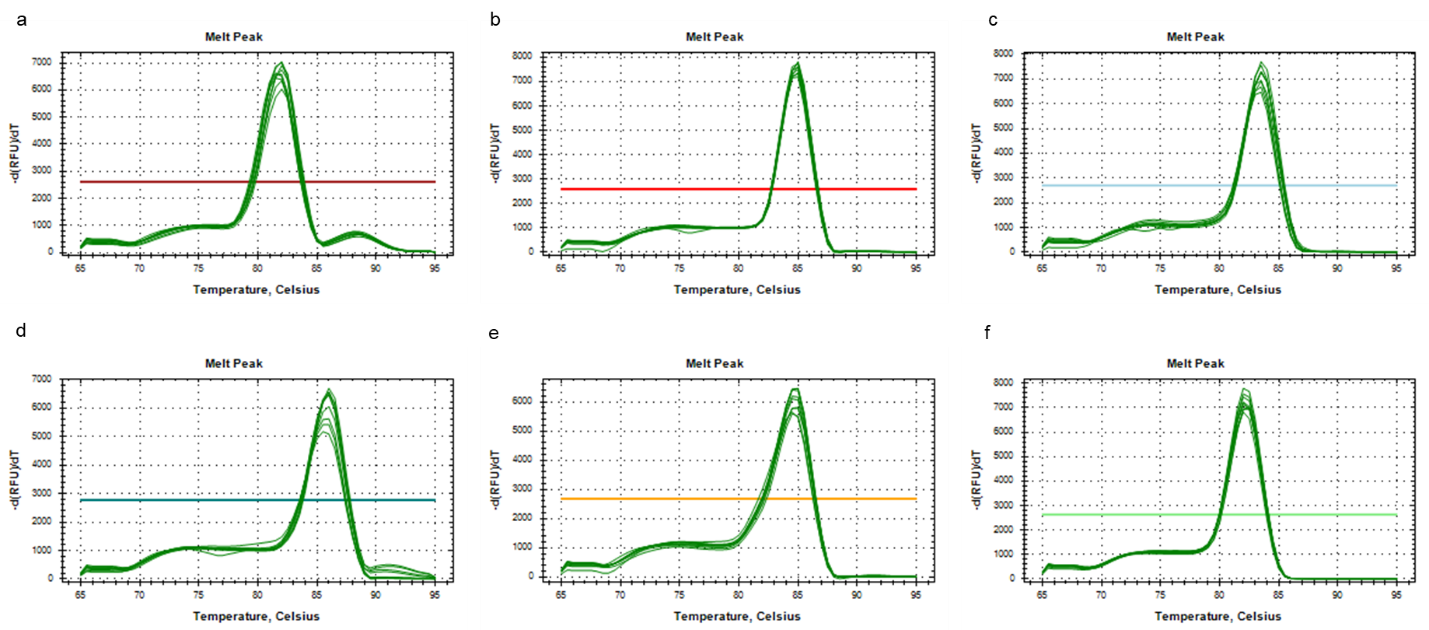


**Figure S2** The melt curve of genes (a) *ldh*; (b) *hcrR*; (c) *hcrA*; (d) *hcrB*; (e) *hcrC*; and (f) *ceh*, during qRT-PCR of *Lact*. *plantarum* SN13T genes encoding housekeeping gene, transcription regulator, NADPH-dependent FMN reductase family protein, NADPH-dependent FMN reductase and hypothetical protein of *hcrABC* operon of reductase genes and cinnamoyl ester hydrolase, respectively, involved in the fermentation of Mentha extract.


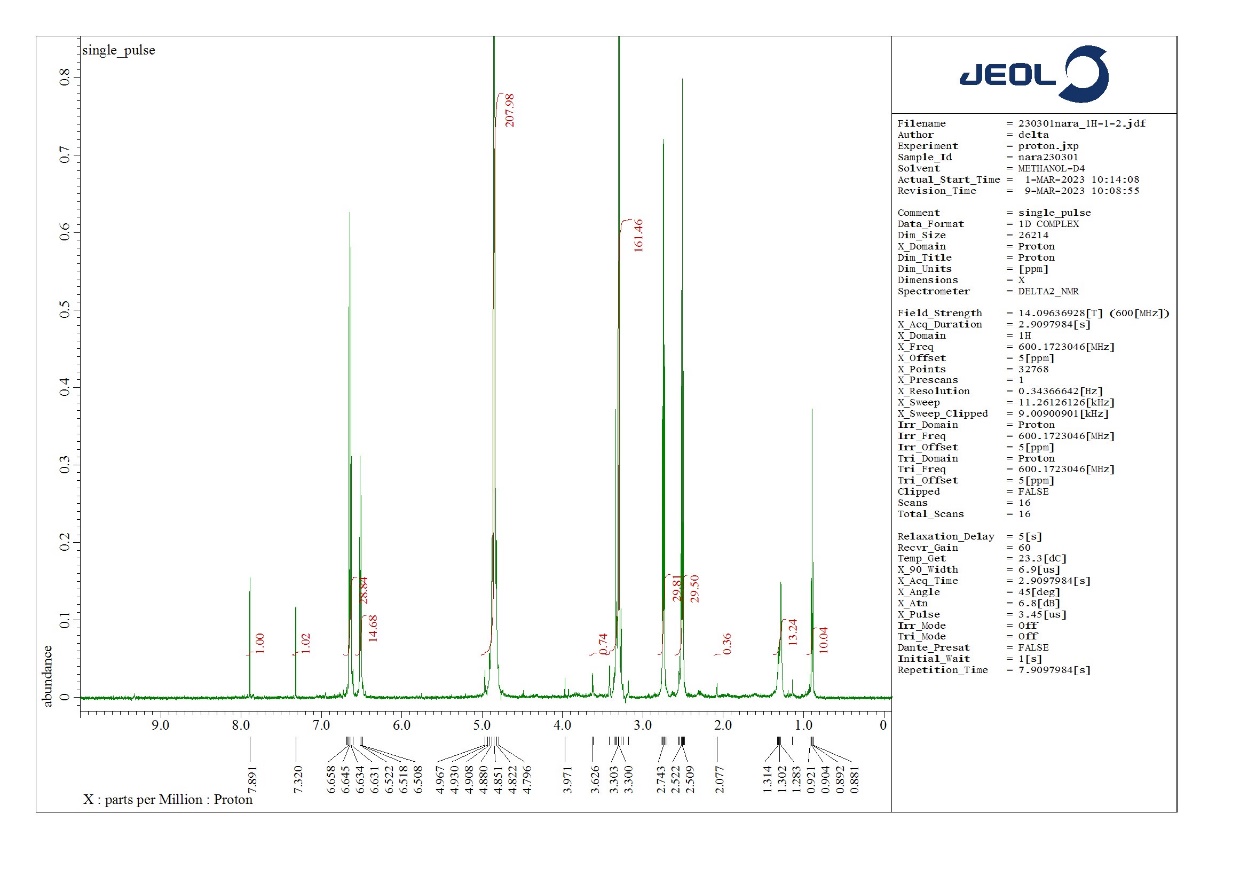


**S3 (b)**

**S3 (a)**


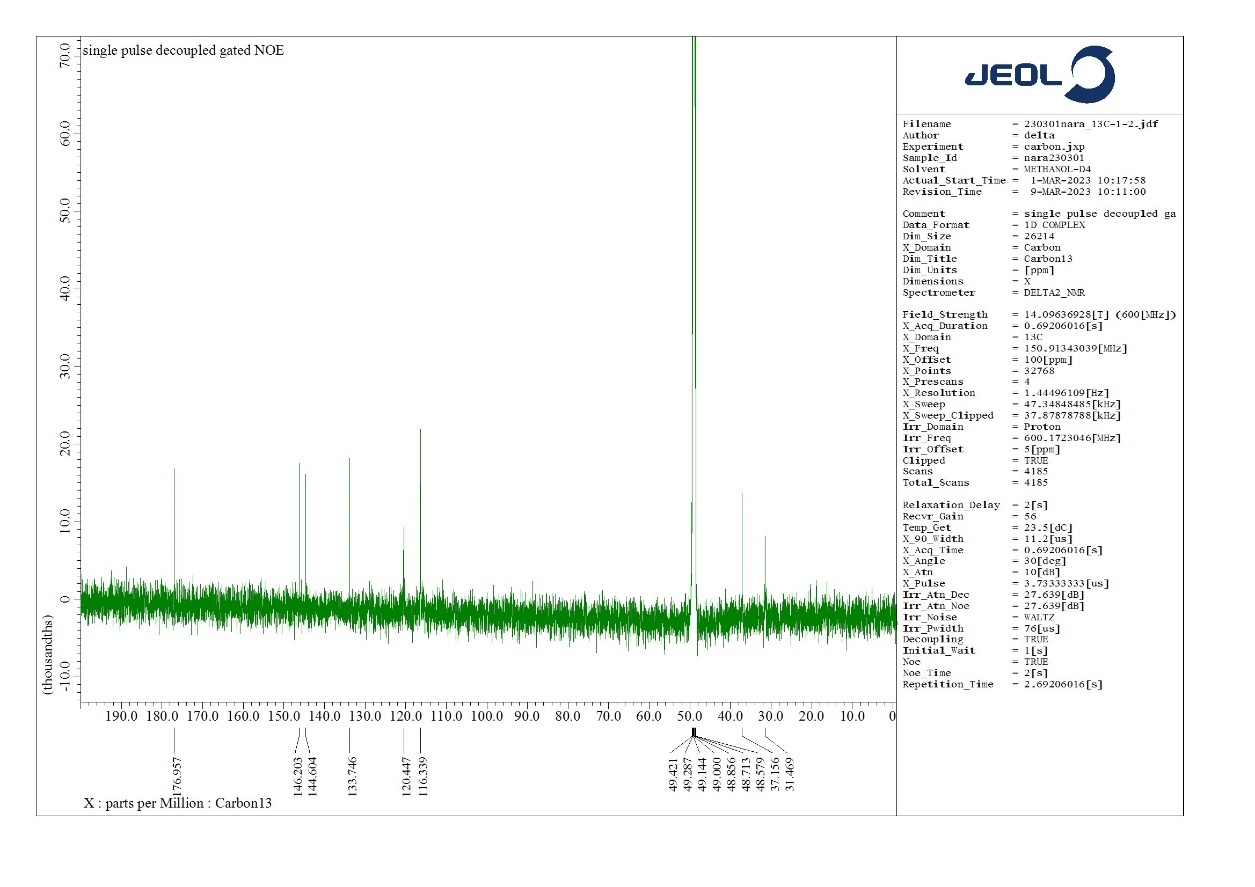


**Figure S3** (a) ^1^H-NMR spectra and (b) ^13^C-NMR spectra of Dihydrocaffeic acid, purified from fMA-SN13T


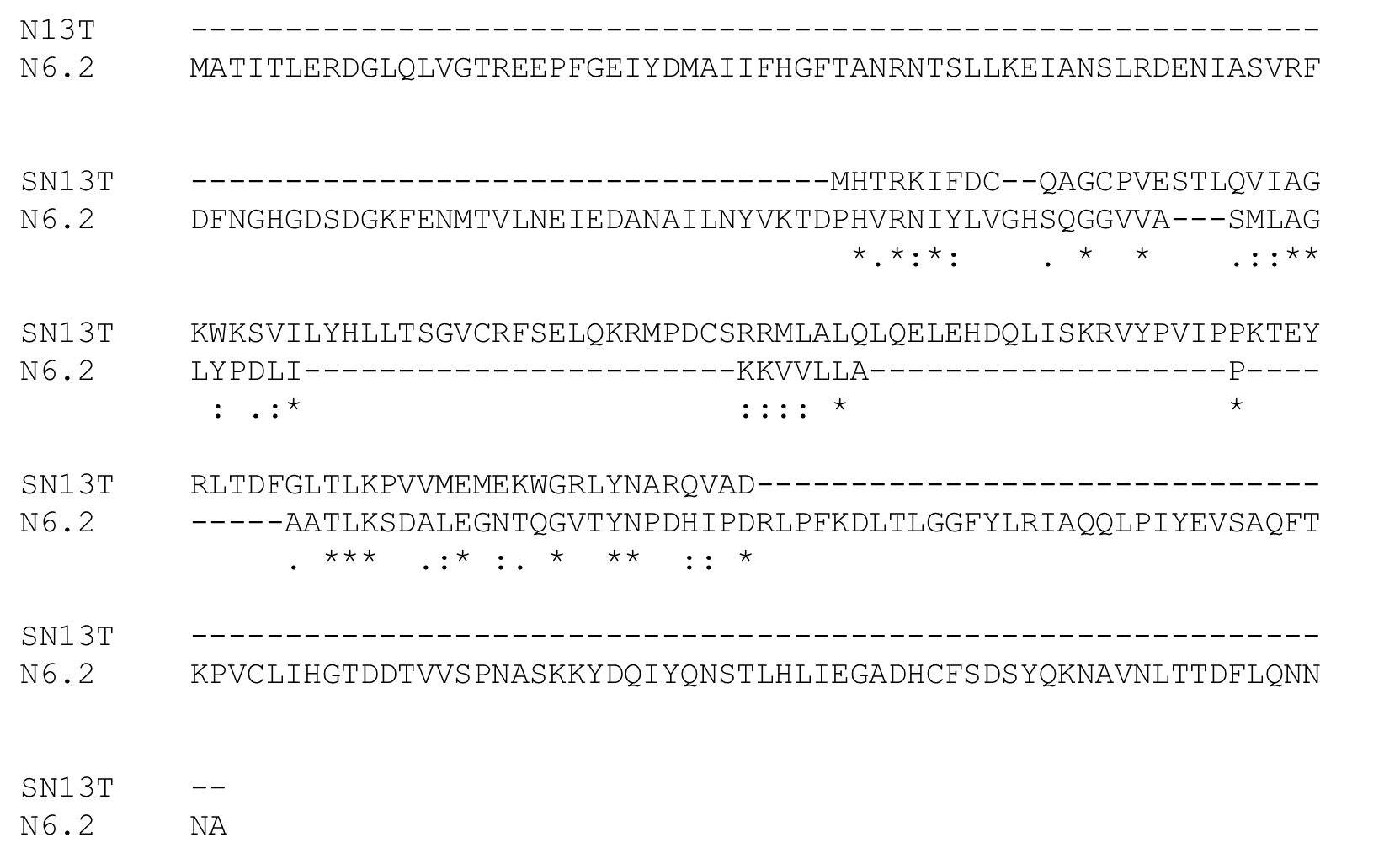


**S4 (b)**

**S4 (a)**


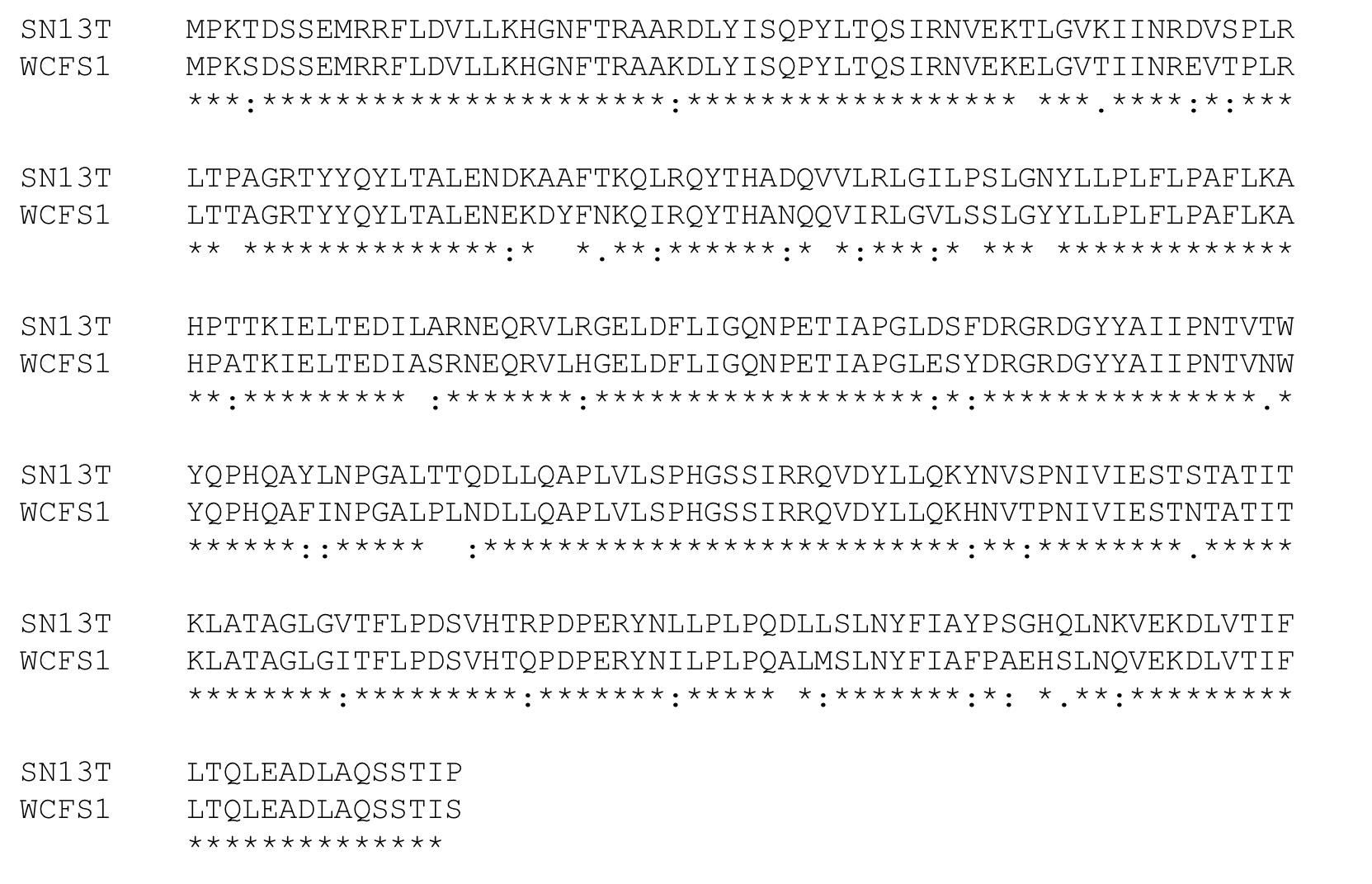


**S4 (c)**


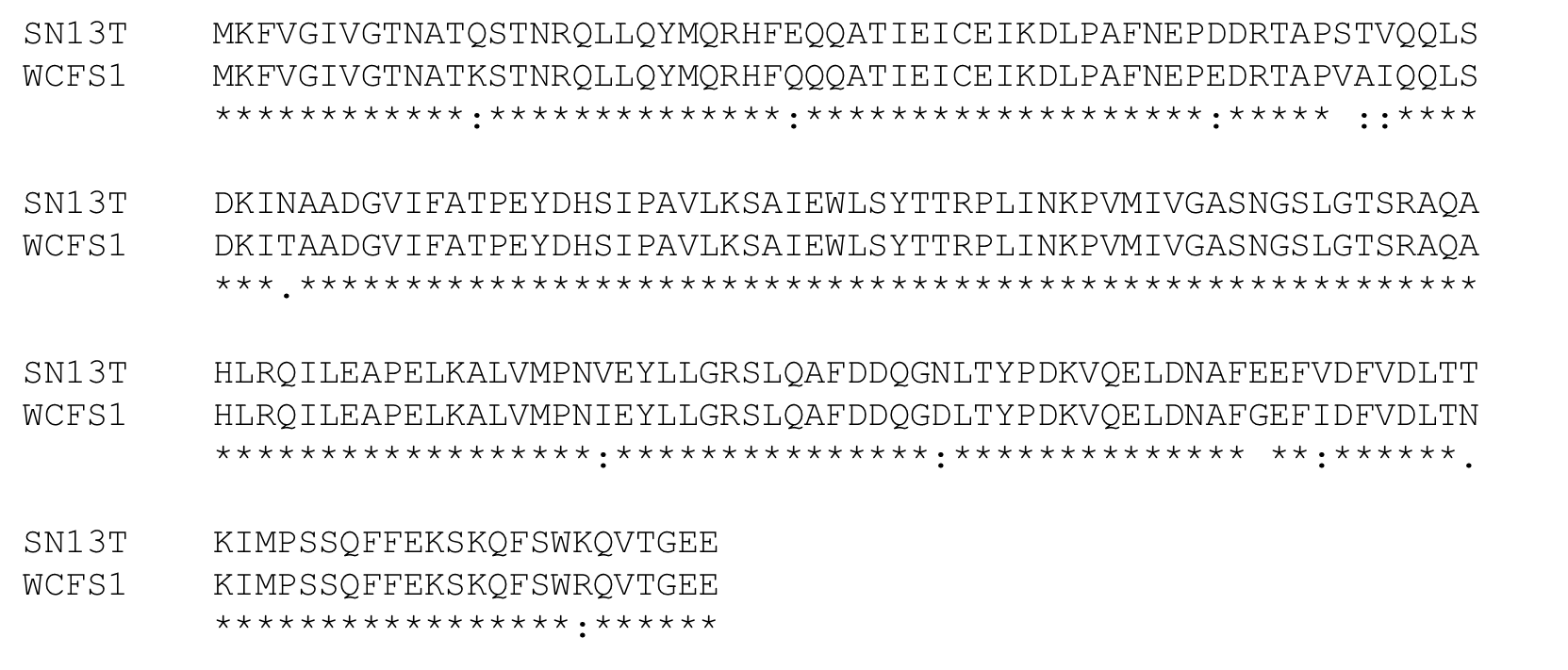


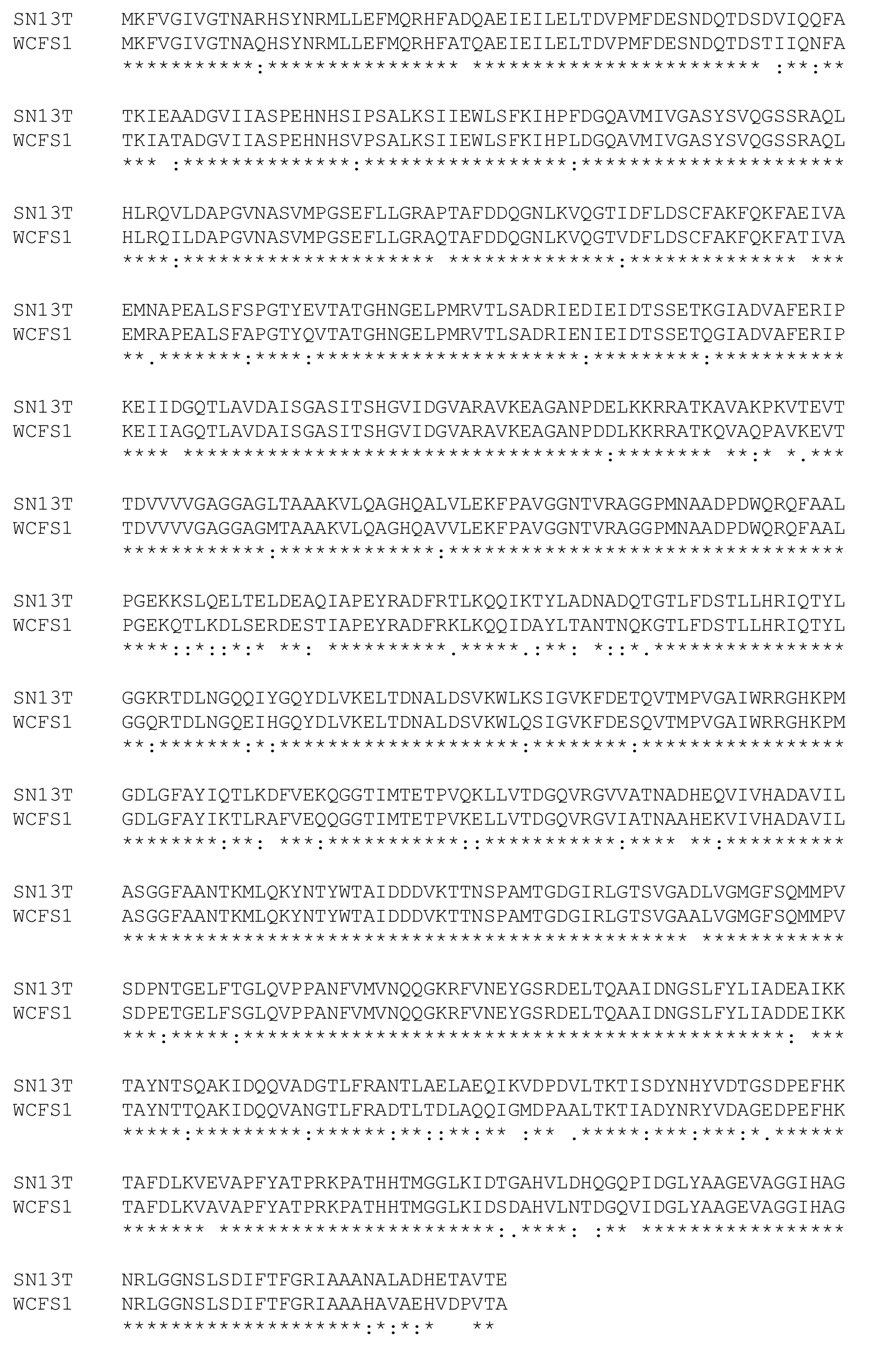


**S4 (d)**

**S4 (e)**


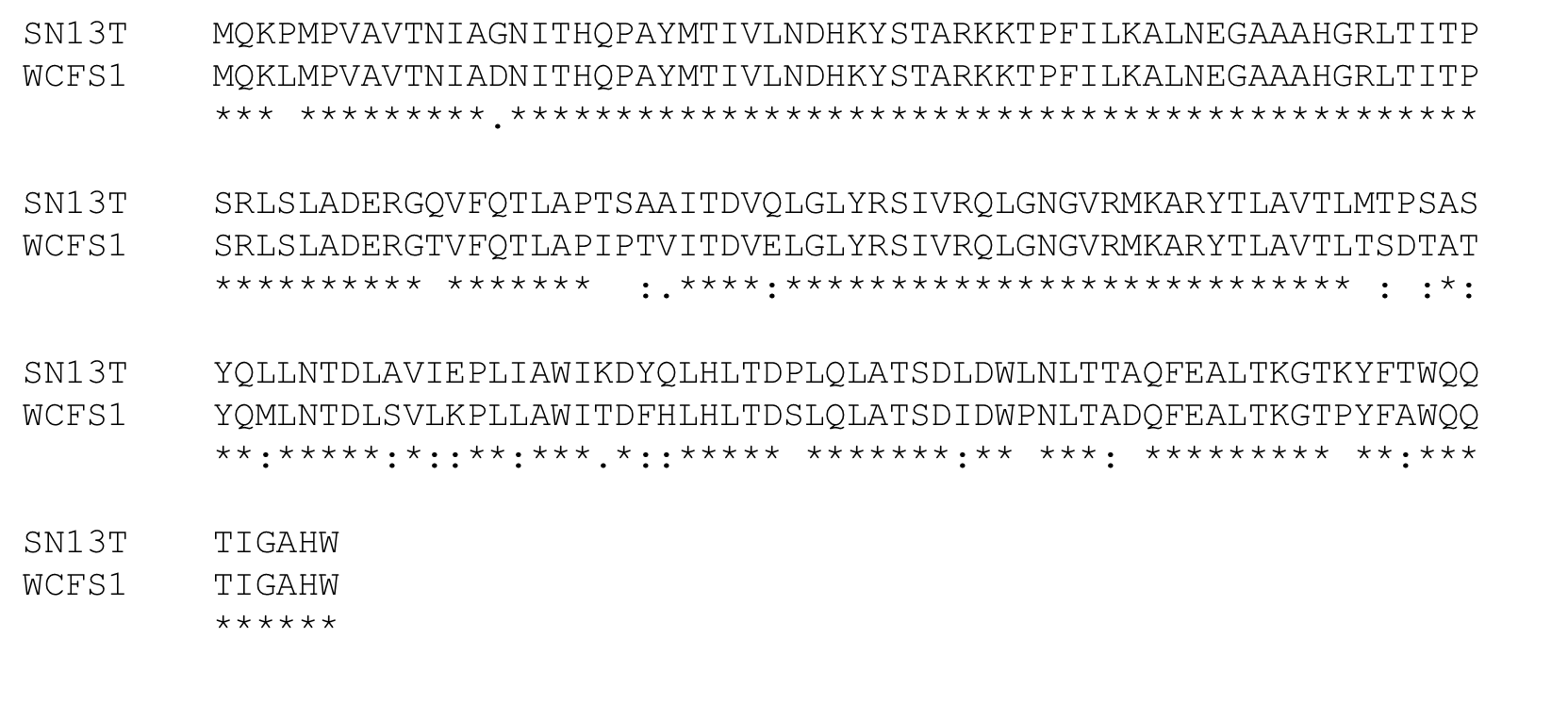


**Figure S4** Comparison of amino acid sequences of putative (a) *ceh* (b) *hcrR,* (c) *hcrA,* (d) *hcrB* and (e) *hcrC.* Multiple alignments were done using the program ClustalOmega after retrieval of sequences from BLAST homology searches. Residues that are identical (*), conserved (:) or semiconserved (.) in all sequences are indicated. Dashes indicated gaps introduced to maximize similarities.

**S5**


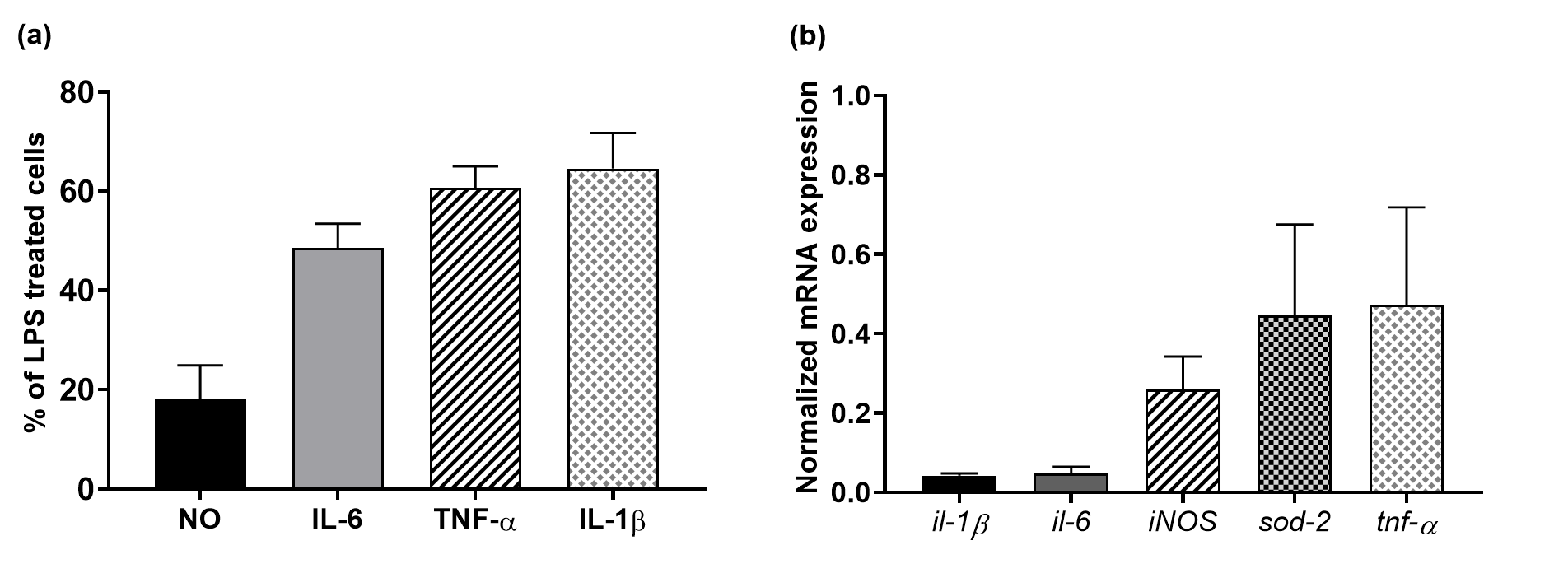


**Figure S5** (a) The concentration of inflammatory mediators NO, IL-6, TNF-α and IL-1β in LPS-stimulated RAW 264.7 cells in presence of Dexamethasone (0.5 µg/ml), expressed as percentage of LPS only treated cells (%); (b) Normalized expressions of inflammatory genes *il-1β, il-6, iNOS, sod-2* and *tnf-α* in LPS-stimulated RAW 264.7 cells in presence of Dexamethasone (0.5 µg/ml). Data are expressed as the mean value of triplicate experiments. Error bars represent +/- standard deviation. All data were significantly different, i.e., p < 0.05 vs LPS treated cells.
